# Anti-inflammatory effect of gold nanoparticles supported on metal oxides

**DOI:** 10.1038/s41598-021-02419-4

**Published:** 2021-11-30

**Authors:** Takashi Fujita, Maeva Zysman, Dan Elgrabli, Toru Murayama, Masatake Haruta, Sophie Lanone, Tamao Ishida, Jorge Boczkowski

**Affiliations:** 1 Department of Applied Chemistry for Environment, Graduate School of Urban Environmental Sciences, Tokyo Metropolitan University, 1-1Minami-osawa, Hachioji, Tokyo, 192-0397 Japan; 2 Department of Applied Chemistry, School of Engineering, Tokyo University of Technology, 1401-1 Katakura, Hachioji, Tokyo, 192-0982 Japan; 3 Univ Paris est Creteil, INSERM, IMRB, 94010 Creteil, France; 4 Univ Bordeaux, Centre de Recherche Cardio-Thoracique de Bordeaux, U1045, CIC, 1401 Bordeaux, France; 5 Service des Maladies Respiratoires, CHU Bordeaux, Bordeaux, France; 6 SAS NaorInnov, Courbevoie, France; 7 AP-HP, Hopital Henri Mondor, Antenne de Pneumologie, 94010 Creteil, France

**Keywords:** Metals, Biophysical chemistry, Nanobiotechnology

## Abstract

Gold (Au) can be deposited as nanoparticles (NPs) smaller than 10 nm in diameter on a variety of metal oxide (MOx) NPs. Au/MOx have high catalytic performance and selective oxidation capacity which could have implications in terms of biological activity, and more specifically in modulation of the inflammatory reaction. Therefore, the aim of this study was to examine the effect of Au/TiO_2_, Au/ZrO_2_ and Au/CeO_2_ on viability, phagocytic capacity and inflammatory profile (TNF-α and IL-1β secretion) of murine macrophages. The most important result of this study is an anti-inflammatory effect of Au/MOx depending on the MOx nature with particle internalization and no alteration of cell viability and phagocytosis. The effect was dependent on the MOx NPs chemical nature (Au/TiO_2_ > Au/ZrO_2_ > Au/CeO_2_ if we consider the number of cytokines whose concentration was reduced by the NPs), and on the inflammatory mediator considered. The effect of Au/TiO_2_ NPs was not related to Au NPs size (at least in the case of Au/TiO_2_ NPs in the range of 3–8 nm). To the best of our knowledge, this is the first demonstration of an anti-inflammatory effect of Au/MOx.

## Introduction

Gold (Au) can be deposited as nanoparticles (NPs) smaller than 10 nm in diameter on a variety of metal oxide (MOx) NPs (Au/MOx) (see Fig. S1 as an example). Au/MOx have attracted much attention due to their high catalytic performance for such as room temperature CO oxidation and selective oxidations in liquid phase^1,2^. The catalytic activity of Au strongly depends on the kind of support. For example, although TiO_2_ is almost inactive for CO oxidation, the deposition of Au NPs onto TiO_2_ enables to catalyze CO oxidation at room temperature^3,4^. The selective oxidation of glucose by Au supported on activated carbon or metal oxides has been a very active research area, as the transformation of readily available glucose to valuable gluconic acid is of great importance^5,6^.

These data in the field of heterogeneous catalysis have firmly established that deposition of Au on MOx NPs increases dramatically the intrinsic catalytic activity and this could also be the case for the biological activity. For example, Menchon et al.^7^ reported that Au/CeO_2_ exhibits antioxidant activity against reactive oxygen species (ROS) in Hep3B and HeLa cell related lines due to a peroxidase activity. Since the inflammatory reaction is highly dependent on oxidative stress^8^ one can hypothesize that, in addition to their antioxidant properties, Au/MOx can have anti-inflammatory effects. Such an effect could have important implications in terms of medical utilization of Au/MOx. However, to the best of our knowledge no data on this effect is available in the current literature.

Therefore, in the present study we examined the effect of Au/MOx on cytotoxic and inflammatory response of macrophages, a key cell type involved in the inflammatory reaction. We used murine macrophages and investigated the roles of the size of Au NPs, the chemical nature of the supporting MOx NPs, and the kinetics of Au/MOx interference with the inflammatory reaction.

## Results

### Physicochemical properties of Au/MOx

**Figure 1:**
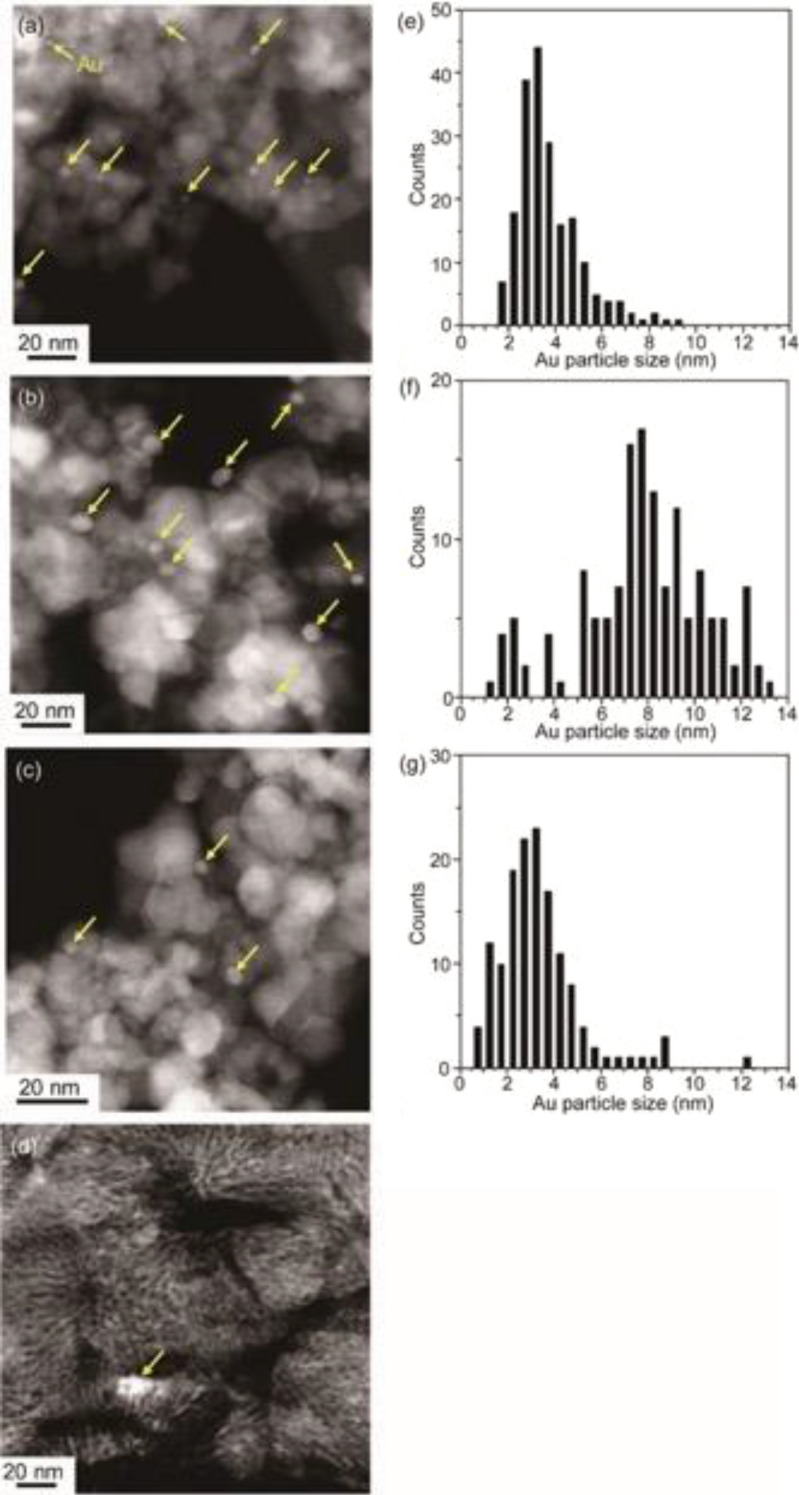
HAADF-STEM images of Au3/TiO_2_ (**a**), Au8/TiO_2_ (**b**), Au/ZrO_2_ (**c**), Au/CeO_2_ (**d**) and their size distributions of Au nanoparticles for Au3/TiO_2_ (**e**), Au8/TiO_2_ (**f**), and Au/ZrO_2_ (**g**). Au nanoparticles were observed as brighter spots and yellow arrows indicate the Au nanoparticles. Only small amount of large Au aggregates was found in Au/CeO_2_.

Physicochemical properties of Au/MOx are summarized in Table 1. Sizes of primary particles of MOx (TiO_2_, ZrO_2_, and CeO_2_) were in the range of 7–26 nm but MOx NPs are generally agglomerated to form secondary micrometer-sized (2.4–9.4 μm) particles. Actual gold loading amounts of Au/MOx were ca. 1 wt% for all samples. The average sizes of Au NPs on TiO_2_ and ZrO_2_ prepared by deposition–precipitation were both of 3.3 nm. To examine the size effect of Au NPs, Au NPs with an average size of 8 nm were deposited on TiO_2_ by solid grinding. High-angle annular dark-field scanning transmission electron microscopic (HAADF-STEM) images of Au/MOx are shown in Fig. 1. For Au/TiO_2_ and Au/ZrO_2_, Au NPs were highly dispersed on MOx NPs. On CeO_2_, the most of Au species is atomically dispersed but only small amount Au NPs with ca. 20 in diameter was observed (Fig. 1d).

**Table 1:** Physicochemical properties of Au NPs supported on metal oxides.

	Specific surface area /m^2^/g^a^	Primary particle size of metal oxide/nm^b^	Secondary particle size of metal oxide/μm^c^	Au loading/wt%^d^	Au particle size/nm^e^
Au3/TiO_2_	54	26	9.4	0.96	3.3 ± 1.4
Au8/TiO_2_	54	26	9.4	1.18	7.8 ± 2.7
Au/ZrO_2_	94	11	3.6	0.97	3.3 ± 1.8
Au/CeO_2_	114	7.3	2.4	0.96	No data^f^

^a^Specific surface area of metal oxide was calculated from N_2_ adsorption isotherms using the Brunauer–Emmett–Teller (BET) method.

^b^Primary particle size of metal oxide was estimated from the specific surface area.

^c^Secondary particle size of metal oxide after the deposition of Au NPs was measured by dynamic laser scattering.

^d^Actual loading amount of Au was determined by inductively coupled plasma-atomic emission spectroscopy (ICP-AES) or atomic absorption spectrometry (AAS).

^e^Average particle size of Au NPs on metal oxides were estimated by HAADF-STEM.

^f^Majority of Au species was considered to be atomically dispersed on CeO_2_ and could not be detected due to a limitation of resolution and low contrast between heavy Ce and Au atoms by TEM.

### No cytotoxic effect of Au/TiO_2_ NPs on mouse macrophages

**Figure 2:**
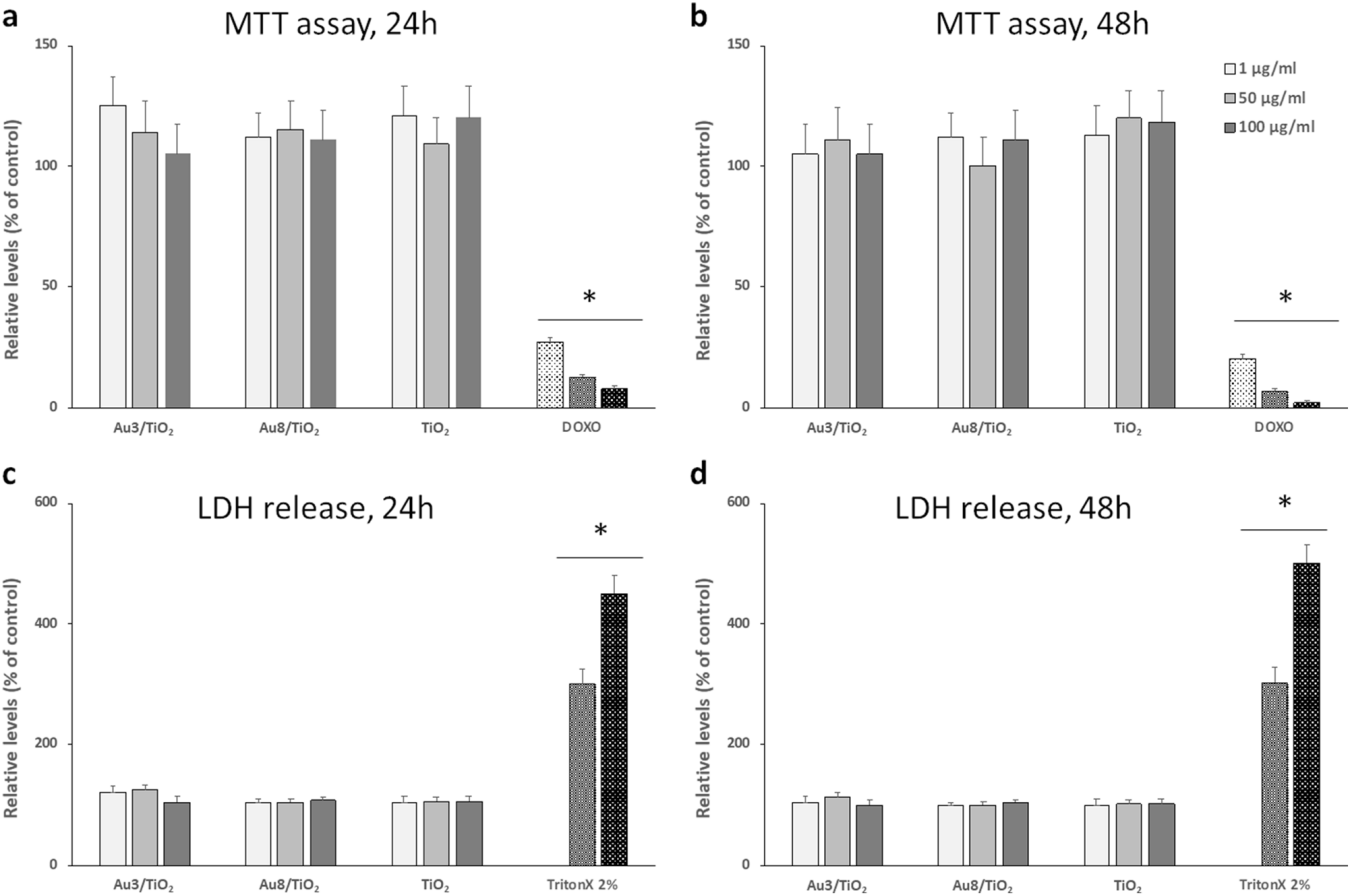
Cytotoxicity of Au nanoparticles (NPs) with two different diameters (3 and 8 nm respectively) supported on pristine TiO_2_ (Au3/TiO_2_ and Au8/TiO_2_ respectively) and TiO_2_ NPs alone on mouse peritoneal macrophages. Cell viability was measured using the MTT assay (**a**,**b**) and LDH relargage (**c**,**d**) after 24 and 48 h incubation (panels a and c, and b and d, respectively). Data are expressed as percentage of control condition (without NPs, 100% is the reference value) and are represented as mean ± standard error of the mean (SEM) of 6 independent experiments. Positive controls are doxorubicine (DOXO, 1, 5 and 10 µg ml^−1^) and TritonX-100 (10 vol. %). * significantly different from control (*p* < *0.05*).

Since Au/TiO_2_ has been extensively studied in the field of catalysis^1,3,4^ we have first compared biological effects of Au/TiO_2_ samples having different size of Au NPs versus TiO_2_ NPs on mouse peritoneal macrophages. Au NPs with two different mean diameters (3 and 8 nm) supported on pristine TiO_2_ NPs were termed Au3/TiO_2_ and Au8/TiO_2_, respectively. No cytotoxicity was observed by either 3-[4,5-dimethylthiazol-2-yl]-2,5 diphenyl tetrazolium bromide (MTT) or lactate dehydrogenase (LDH) release assays for TiO_2_ and Au/TiO_2_ NPs regardless of the concentration (1 to 100 µg ml^−1^), the exposure time (24 and 48 h) and the size of Au NPs (Fig. 2).

In addition to cytotoxicity evaluation, we examined whether Au/TiO_2_ NPs modified the phagocytic capacity of macrophages (an important function of these cells) as compared to TiO_2_ NPs. This was performed by quantifying phagocytosis of fluorescent beads after cells incubation with TiO_2_ and Au/TiO_2_ NPs (50 µg ml^−1^, 6 h incubation). The results of these experiments showed both TiO_2_ and Au/TiO_2_ NPs significantly increased the phagocytosis of fluorescent beads by peritoneal macrophages, but the differences between TiO_2_ and Au/TiO_2_ was not observed. This result shows that Au NPs did not modulate phagocytic capacity of macrophages (an important function of these cells).

Macrophages incubation with TiO_2_ and Au/TiO_2_ NPs significantly and similarly increased the phagocytosis of fluorescent beads by peritoneal macrophages (Fig. S2).

### TiO_2_ and Au/TiO_2_ NPs are internalized in mouse peritoneal macrophages

Cellular uptake and intracellular morphology upon 24 h exposure to TiO_2_ and Au/TiO_2_ NPs were investigated in ultra-thin sections of resin-embedded cells using TEM.

TiO_2_ NPs and Au/TiO_2_ NPs (50 µg ml^−1^) were internalized in cells. The prevalent localization of NPs agglomerates was observed in cell vacuoles (Fig. S3). In accordance with the previous paragraph showing a lack of cytotoxicity, no morphologic sign of cell damage was observed.

### No inflammation induced by Au/TiO_2_ NPs

Incubation of mouse peritoneal macrophages with TiO_2_ NPs (50 µg ml^−1^, 6 h), induced a significant increase in the concentration of TNF-α and IL-1β, two main pro-inflammatory cytokines, in cell culture medium (Fig. 4). In contrast, the deposition of Au NPs onto TiO_2_ greatly reduced the concentration of both TNF-α and IL-1β as compared to pristine TiO_2_, and this effect was independent on the size of Au NPs.

### Au/TiO_2_ NPs attenuate lipopolysaccharide (LPS)-induced inflammation

Having demonstrated that Au/TiO_2_ NPs did not elicit an inflammatory reaction, we questioned whether Au/TiO_2_ NPs could blunt inflammation induced in a model of pathophysiological relevance, such as macrophages exposure to *Escherichia Coli* LPS. Since no difference of anti-inflammatory effect was observed between Au3/TiO_2_ and Au8/TiO_2_, we investigated the effects of Au3/TiO_2_ NPs (referred to as Au/TiO_2_ in the following experiments). Mouse peritoneal macrophages were incubated with culture media for 6 h (control condition) or LPS for 2 h, and then the following 4 h with LPS alone or LPS plus TiO_2_ or Au/TiO_2_. Taking into account the results presented in the previous paragraph, TNF-α and IL-1β cell culture supernatant concentrations were measured as a surrogate of the inflammatory reaction. As expected, incubation of cells with LPS for 2 + 4 h induced a significant increase as compared to the control condition (p < 0.05, Fig. 5a,b). Incubation of LPS followed by TiO_2_ NPs did not significantly modify the effect of LPS alone. By contrast, this last effect was significantly attenuated by macrophage incubation with Au/TiO_2_ NPs (p < 0.05, Fig. 5a,b. These results show that Au/TiO_2_ can attenuate LPS induced inflammation, even when cells are incubated with these NPs after incubation with LPS.

### Effects of Au/ZrO_2_ and Au/CeO_2_ NPs on LPS-induced inflammation

**Figure 3:**
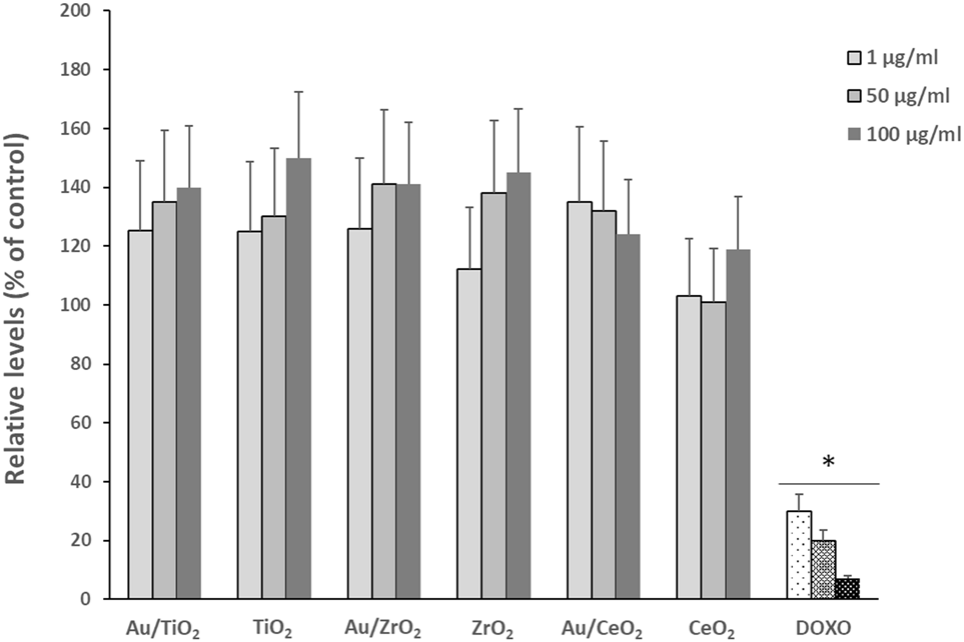
Cytotoxicity of Au nanoparticles (NPs) supported on TiO_2_, ZrO_2_ and CeO_2_ NPs and TiO_2_, ZrO_2_ and CeO_2_ NPs alone on mouse peritoneal macrophages. Cell viability was measured using the WST-1 assay after 24 h incubation. Data are expressed as percentage of control condition (without NPs, 100% is the reference value) and are represented as mean ± SEM of 6 independent experiments. Positive control is doxorubicine (DOXO, 1, 5 and 10 µg ml^−1^) * significantly different from control (*p* < *0.05*).

After showing the anti-inflammatory effect of Au/TiO_2_ NPs, we investigated if this effect was related to the chemical nature of the MOx NPs. To answer this question, we examined the effect of Au NPs deposited on either ZrO_2_ or CeO_2_ NPs on mouse peritoneal macrophages. Physicochemical characterization of these Au/MOx are provided in Table 1. No effect of these NPs on macrophages viability was observed (Fig. 3).

Concerning LPS-induced inflammation, no effect was observed with Au/CeO_2_ NPs, whereas Au/ZrO_2_ NPs attenuated LPS-induced IL-1β production without any effect on TNF-α concentration (Fig. 5a,b). Therefore, we can conclude that Au/MOx possess an anti-inflammatory effect which depends on the nature of the MOx NPs and on the inflammatory mediator examined.

### Is the anti-inflammatory effect of Au/MOx NP related to cytokines adsorption on their surface?

We investigated the mechanisms underlying the anti-inflammatory effect of Au/MOx. Since MOx NPs adsorb different proteins on their surfaces^9^, we then investigated if the decreased concentration of LPS-induced TNF-α and IL-1β concentration observed with Au/TiO_2_ and Au/ZrO_2_ (only for IL-1β in this last case) was related to adsorption of these cytokines on these Au/MOx. The results of these experiments showed that incubation of each cytokine with the different Au/MOx did not change its respective concentration when compared to the MOx NPs without Au (Fig. 6). This result excludes cytokines adsorption as a mechanism explaining the anti-inflammatory effect of Au/TiO_2_ and Au/ZrO_2_.

### Is the anti-inflammatory effect of Au/MOx related to their antioxidant properties?

We then examined if the anti-inflammatory effect of Au/TiO_2_ and Au/ZrO_2_ was related to antioxidant properties, as demonstrated in other conditions^10,11^. To investigate this issue, we measured the effect of Au/MOx NPs on intracellular reactive oxygen species (ROS) concentration. These experiments showed that intracellular ROS production induced by Au/TiO_2_ and Au/ZrO_2_ NPs was smaller than that of TiO_2_ and ZrO_2_ respectively, demonstrating an antioxidant effect of Au/TiO_2_ and Au/ZrO_2_ (Table 2), in accordance with their (partial) anti-inflammatory effect. This effect was more pronounced in the case of Au/ZrO_2_ compared to Au/TiO_2_ (mean reduction of 32% vs 11% compared to ZrO_2_ and TiO_2_ respectively). This antioxidant effect could be involved in the anti-inflammatory effect described previously. Interestingly, the level of ROS in cells exposed to CeO_2_ was lower as compared to those in cells exposed to TiO_2_ and ZrO_2_, which is in accordance with antioxidant properties of CeO_2_ described previously^12^. Since the pristine CeO_2_ NPs possess antioxidant properties, the effect of Au deposited on CeO_2_ was probably limited.

**Table 2:** Reactive oxygen species (ROS) production in RAW 264.7 cells after expositions to different NPs.

Nanoparticle	Mean values ± SEM	Statistical significance
Au/TiO_2_	12,068 ± 499	*
TiO_2_	13,547 ± 340	
Au/ZrO_2_	9170 ± 109	**
ZrO_2_	13,538 ± 129	
Au/CeO_2_	12,743 ± 340	
CeO_2_	12,583 ± 255	#

The final concentration of NPs was 50 µg ml^−1^. Amounts of ROS production were evaluated by the DCFH-DA (n = 7).

*p < 0.05 vs TiO_2_, **p < 0.01 vs ZrO_2_, ^#^*p* < 0.05 vs TiO_2_ and ZrO_2_.

## Discussion

**Figure 4:**
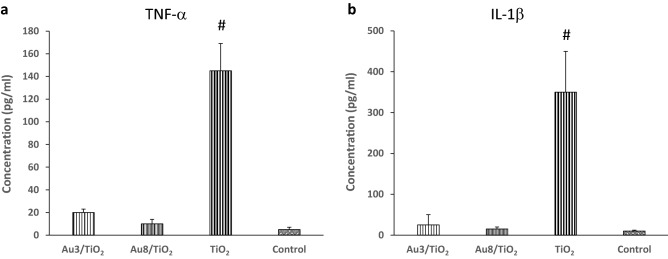
Effect of Au3/TiO_2_, Au8/TiO_2_ and TiO_2_ NPs (50 µg ml^−1^, 6 h incubation) on TNF-α and IL-1β secretion by mouse peritoneal macrophages (**a**,**b**). Data are expressed as picograms/ml in cell culture medium and are represented as mean ± SEM of 6 independent experiments. ^#^Significantly different from control (*p* < 0.01).

**Figure 5:**
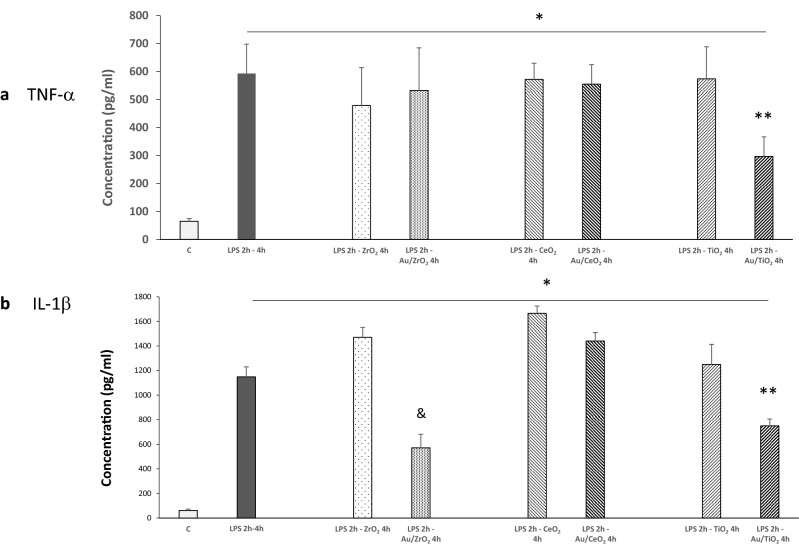
Effect of Au deposited on different metal oxide NPs on LPS induced TNF-α and IL-1β secretion by mouse peritoneal macrophages (**a**,**b**). Cells were incubated with culture media for 6 h (control condition, C) or LPS for 2 h, and then the following 4 h with LPS alone or LPS plus the different MOx or Au/MOx NPs (50 µg ml^−1^). *Significantly different from C (*p* < 0.05), **significantly different from LPS 2 h—TiO_2_ 4 h (*p* < *0.05*), ^&^significantly different from LPS 2 h—ZrO_2_ 4 h (*p* < 0.05).

The most important result of this study is an anti-inflammatory effect of Au/MOx NPs depending on the MOx nature with particle internalization but no alteration of cell viability or phagocytic capacity of Au/MOx NPs as compared to MOx NPs (Figs. 2 and 3). The effect of Au/TiO_2_ NPs was not related to Au NPs size (at least in the case of Au/TiO_2_ NPs in the range of 3–8 nm) (Fig. 4) and was dependent on the MOx NPs chemical nature (Au/TiO_2_ > Au/ZrO_2_ > Au/CeO_2_ if we consider the number of cytokines whose concentration was reduced by the NPs) and on the inflammatory mediator considered (Fig. 5). To the best of our knowledge, this is the first demonstration that Au/MOx presents anti-inflammatory properties.

The effect of Au/MOx could be related to anti-inflammatory properties of Au NPs per se. Indeed, gold colloids have been thought to cure various diseases for many centuries, and recent studies have demonstrated that this effect is mediated by inhibition of NF-κB activation^13^. Different studies showed a similar effect in the case of Au NPs in vitro and in vivo, this effect being usually mediated by antioxidant properties^14–16^. However, other studies showed opposite results. For example, Ng et al.^16^ showed that incubation of human bronchial epithelial cells with 50 µg ml^−1^ of 20 nm Au NPs activated NF-κB in bronchial epithelial cells. Close results were shown in other cell types^17,18^. Therefore, an anti-inflammatory effect of Au NPs per se seems not a univocal mechanism explaining the anti-inflammatory effect of Au/MOx NPs. Moreover, most of the studies showing the effect of Au NPs on inflammation have been conducted at much higher concentrations than the cells were exposed to in this study. Considering that Au NPs account for only 1% of the mass of Au/MOx, simply attributing the present results to the biological activity of Au NPs is unlikely.

**Figure 6:**
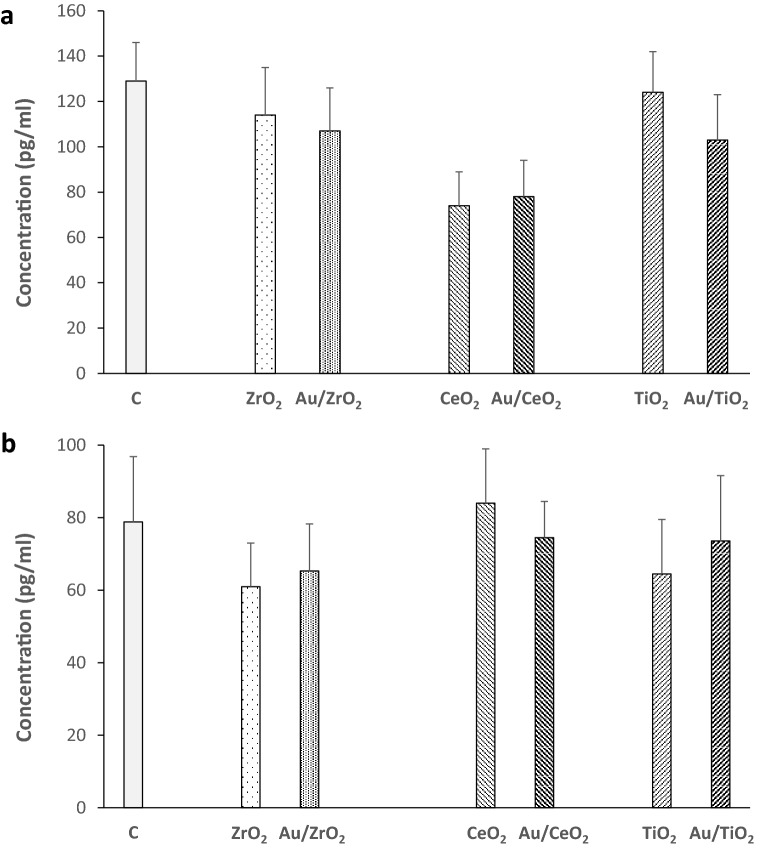
TNF-α and IL-1β adsorption on Au deposited on different MOx NPs. MOx or Au/MOx NPs (50 µg ml^−1^) were incubated during 6 h with TNF-α (150 pg ml^−1^) and IL-1β (100 pg ml^−1^) in culture media, then the cytokines were quantified in the media after centrifugation.

Adsorption of TNF-α and IL-1β on Au/MOx surface could also explain their different anti-inflammatory activity. Indeed, NPs, including Au NPs, can adsorb different molecules on their surface, resulting in their inactivation and reduction in their concentration in the medium^19^. However, this mechanism seems unlikely in the present study, since we did not observe any significant difference in TNF-α and IL-1β levels when these cytokines were incubated with TiO_2_ or ZrO_2_ NPs and their respective Au counterparts (Fig. 6).

Finally, the differences in the anti-inflammatory effect of Au/MOx NPs could be related to their different antioxidant properties. However, the anti-inflammatory effect of Au/MOx NPs was not good consistent with the intracellular antioxidant properties, since both Au/TiO_2_ and Au/ZrO_2_ NPs reduced intracellular ROS concentration whereas only Au/TiO_2_ attenuated both TNF-α and IL-1β induction (Fig. 5). In concordance with different studies, CeO_2_ NPs exerted an antioxidant effect as compared to TiO_2_ and ZrO_2_ NPs (Table 1), but this effect was not accompanied by an anti-inflammatory effect. On the other hand, the absence of an anti-inflammatory effect of Au/CeO_2_ was paralleled by an absence of an antioxidant effect of CeO_2_ NPs. Collectively, these data suggest that, in contrast with our initial hypothesis, an antioxidant effect is not a mechanism globally explaining the anti-inflammatory effect of Au/TiO_2_ and Au/ZrO_2_. Indeed, an antioxidant effect could potentially explain the effect of Au/TiO_2_ NPs but not the one of Au/ZrO_2_ NPs, because Au/ZrO_2_ NPs had showed an antioxidant effect similar to Au/TiO_2_ but attenuated only IL-1β induction. Consequently, the anti-oxidant effect of Au/ZrO_2_ appears to relate on pathways inhibiting IL-1β but not TNF-α expression.

Numerous mechanisms have been reported to control the production and activity of IL-1β, including the processing of the 31-kDa, inactive IL-1β precursor into the bioactive, 17-kDa cytokine via intracellular protein complexes termed the inflammasomes^20^. The most intensely studied inflammasome is the NLRP3 inflammasome. Various stimuli activate the NLRP3 inflammasome: bacterial structures such as muramyl dipeptide or LPS, bacterial RNA, β-glucan, double-stranded RNA, etc. The release of ROS has been reported to mediate NLRP3 inflammasome activation by various stimuli, including LPS, but this has been surrounded by controversy^21^. Other proposed mechanisms responsible for NLRP3 inflammasome activation are: (i) translocation to mitochondria^22^, (ii) release of mitochondrial DNA or cardiolipin^23^, (iii) release of lysosomal cathepsins into the cytoplasm^24^, and (iv) calcium-dependent phospholipase 2 activation^20^. Interference with one or several mechanisms could explain attenuation of LPS-induced IL-1β expression by Au/ZrO_2_ NPs. However, investigating these possibilities needs further studies.

This study presents four main limitations. First, as MOx NPs are made of different materials, the cells will be exposed to a different amount of particles. However, we consider this possibility unlikely because Au content in the different Au/MOx NPs was very low (around 1 wt%). Second, we used two different murine macrophages populations: primary cultures of peritoneal macrophages and the RAW 264.7 macrophages cell line. We chose to use primary cultures of peritoneal macrophages because a large body of literature combining both in vitro approaches using peritoneal macrophages, and in vivo experiments in mice, demonstrated the relevance of the findings obtained in peritoneal macrophages, and their potential translation to what may occur in vivo^25–27^. Moreover, we wanted to be at most in accordance with the 3R's guidelines, and for this reason we performed the experiments with the murine macrophage cell line RAW 264.7. Indeed, as demonstrated by Maurya and coworkers^28^, RAW264.7 cells behave similarly to primary cultures of peritoneal macrophages cells in some ways and can be used as a good model for inflammation- and immune function-related kinetic studies. RAW 264.7 behave different from peritoneal macrophages in other aspects of lipid metabolism and phenotypes used as models for various disorders such as atherosclerosis, but these types of responses were not concerned in the present study. Third, we only measured two cytokines as a surrogate of inflammatory markers. Even if these cytokines are broadly representative of the inflammatory reaction and are one of the very early ones in the inflammatory cascade, and we investigate primary murine macrophages (a key cell involved in inflammation), a wider analysis of mediators of this reaction is necessary to validate the present results. Fourth, we investigated only three MOx NPs supports. Although we choose supports widely characterized in terms of catalytic properties when combined with Au NPs, investigating a broader panel of MOx could allow a better characterizing the anti-inflammatory properties of Au/MOx NPs.

In conclusion, despite of the above-mentioned limitations, this study showed that Au/MOx possess anti-inflammatory properties in macrophages and the effect depended on the kind of MOx NPs. Au/TiO_2_ showed anti-inflammatory effect for both TNF-α and IL-1β. Although Au/ZrO_2_ was ineffective to reduce the concentration of TNF-α, the anti-inflammatory effect for IL-1β was more pronounced for Au/ZrO_2_ as compared to Au/TiO_2_. In addition, Au/TiO_2_ and Au/ZrO_2_ exhibited improved antioxidant properties in RAW 264.7 compared to the pristine TiO_2_ and ZrO_2_ NPs. Although the relationships between antioxidant properties and anti-inflammatory effect of Au/MOx NPs and the mechanism are still unclear, our finding, anti-inflammatory effect of Au/MOx NPs could have potential applications in the clinical setting, since it does not interfere with physiological parameters of these cells such as viability and phagocytosis.

## Methods

### Preparation of Au nanoparticles supported on metal oxides (Au/MOx)

#### Materials

TiO_2_ (AEROXIDE P25) was supplied from Nippon Aerosil Co., Ltd. ZrO_2_ (RC-100) and CeO_2_ (high purity fine cerium oxide) were purchased from Daiichi Kigenso Kagaku Kogyo Co., Ltd. Tetrachloroauric acid (HAuCl_4_·4H_2_O) and dimethyl gold(III)(acetylacetonate), Me_2_Au(acac), were purchased from Tanaka Kikinzoku Kogyo K.K. and Tri Chemical Laboratories Inc., respectively. Au/ZrO_2_ and Au/CeO_2_ prepared by deposition–precipitation were purchased from Haruta Gold Inc. All reagents and materials were used as received.

#### Characterization of Au/MOx

Average particle size of metal oxides after the deposition of Au NPs were measured by laser diffraction/scattering particle size distribution analyzer using a HORIBA, Partica LA-950V2. Specific surface area of metal oxides was estimated from the N_2_ adsorption measurement was carried out using a SHIMADZU, Tristar 3000. The samples were pretreated at 200 °C for 2 h under vacuum, and then the N_2_ adsorption isotherms were obtained at − 196 °C. The Brunauer–Emmet–Teller (BET) method was used to calculate the specific surface area. Loading amount of Au on metal oxides were measured by inductively coupled plasma atomic emission spectroscopy (ICP-AES, Thermo Fisher Scientific, iCAP6500) or atomic absorption spectrometry (AAS, SHIMADZU, AA-6200). The samples were treated in aqua regia to dissolve Au NPs, filtered, and then diluted to measure. Average size of Au NPs supported on metal oxides were estimated by high-angle annular dark-field scanning transmission electron microscopic (HAADF-STEM) observation using a JEOL, JEM-3200FS.

#### Preparation of Au/MOx

Au NPs with two different mean diameters (3 and 8 nm) supported on pristine TiO_2_ NPs were prepared, termed Au3/TiO_2_ and Au8/TiO_2_, respectively. Au3/TiO_2_ was prepared by deposition–precipitation^3^. An aqueous solution of HAuCl_4_ (1 mM, 50 mL) was warmed to 70 °C, and the pH was adjusted to 6 by adding NaOH aqueous solution. TiO_2_ (1.0 g) was added to the solution and the suspension was stirred at 70 °C for 1 h. The suspension was centrifuged, washed with water at 40 °C for five times, and then filtered. The resultant powder was dried in air at 100 °C overnight and calcined at 300 °C for 4 h to reduce Au(III) to Au(0). Au/ZrO_2_ and Au/CeO_2_ were also prepared by deposition–precipitation as well as Au3/TiO_2_. Au8/TiO_2_ was prepared by solid grinding^5^. TiO_2_ (1.0 g) and Me_2_Au(acac) (15 mg) were ground in an agate mortar in air at room temperature for 20 min. The mixture was calcined at 300 °C for 2 h to give Au8/TiO_2_.

Physicochemical properties of the obtained Au/MOx are summarized in Table 1. The primary particle sizes of Au/TiO_2_, Au/ZrO_2_, and Au/CeO_2_ were 26 nm, 11 nm, and 7.3 nm respectively, estimated from their specific surface areas of the MOx NPs. The secondary particle sizes of Au/TiO_2_, Au/ZrO_2_, and Au/CeO_2_ were 9.4 µm, 3.6 µm, and 2.4 µm, respectively, determined by light scattering. The actual Au loadings for Au3/TiO_2_, Au8/TiO_2_, Au/ZrO_2_, and Au/CeO_2_ were 0.96, 1.18, 0.97, and 0.96 wt%, respectively. The mean diameters of Au NPs were estimated by HAADF-STEM observation to be 3.3 ± 1.4 nm for Au3/TiO_2_, 7.8 ± 2.7 nm for Au8/TiO_2_, and 3.3 ± 1.8 nm for Au/ZrO_2_ (Fig. 1). In the case of Au/CeO_2_, it was difficult to observe Au NPs because Au was mostly deposited as single atoms and the atomic number of Ce is close to that of Au.

The different NPs were evaluated for LPS contamination (Limulus Amebocyte Lysate test), and the observed absorbance at 405 nm was below detection limits demonstrating no LPS contamination.

### Cells isolation and incubation with nanoparticles

Peritoneal macrophages and macrophages from the RAW 264.7 cell line were used in the different experiments.

Mice peritoneal macrophages were prepared as follows. C57BL/6 mice were injected intraperitoneally with 1 ml of 3% Brewer thioglycollate (Sigma-Aldrich, B2551). After 96 h, peritoneal cells were harvested by lavage with 0.67% phosphate buffered saline (PBS). After a soft centrifugation, cells were maintained in Dulbecco’s modified Eagle medium (DMEM) 4.5 g l^−1^ glucose supplemented with 10% fetal calf serum (FCS)and 1% penicillin and 1% streptomycin. Then mice peritoneal macrophagic cells were exposed for 6–48 h to 1–100 µg ml^−1^ particles of Au/TiO_2_, Au/ZrO_2_, Au/CeO_2_, and MOx alone.

The RAW 264.7 cells were cultured using a medium having the same composition as above.

Nanoparticle interference with the signal was tested in all of the colorimetric assays. No interference was observed.

### Cellular viability

The viability of cells was measured by 3-[4,5-dimethylthiazol-2-yl]-2,5 diphenyl tetrazolium bromide (MTT) assay, 2-(4-iodophenyl)-3-(4-nitrophenyl)-5-(2,4-disulfophenyl)-2H-tetrazolium, monosodium salt (WST-1) assay, and the quantification of the release of lactate dehydrogenase (LDH).

### Inflammatory responses

Inflammatory response was evaluated by the quantification of cytokines such as tumor necrosis factor (TNF)-α and interleukin (IL)-1β in cell supernatant, measured by enzyme-linked immunosorbent assay (ELISA).

### Measurement of oxidative stress by DCFH‐DA assay

Endogenous ROS were quantified by oxidation of 2′,7′-dichlorofluorescin diacetate (DCFH‐DA) into 2′,7′-dichlorofluorescin (Sigma, Saint Quentin Fallavier, France). Briefly, cells were cultivated in six‐well culture plates and treated with 50 µg ml^−1^ NPs. Cells were also treated with 250 µM H_2_O_2_ as a positive control (data not shown). Cells were incubated with 20 µM DCFH‐DA for 30 min at 37 °C and fluorescence recorded for 90 min. Results were expressed as the mean ratio of fluorescence recorded every 15 min during the 90 min period.

### Measurement of TNF-α and IL-1β adsorption to NPs

Adsorption of TNF-α and IL-1β adsorption to NPs was performed by ELISA as described previously described^29^.

### Statistical analysis

JASP software (version 0.11.1, https://jasp-stats.org/) was used to analyze quantitative data. Non-parametric ANOVA was used to compare multiple groups. Paired comparisons with Mann–Whitney test were performed if the differences using ANOVA were statistically significantly different. Data were presented as mean values ± standard error of the mean (SEM), and results were considered statistically significant if *p* < *0.05*.

### Study approval for animal experiments

The Institutional Animal Care and Use Committee approved experimental procedures on mice (APAFIS authorization #14914-2018042515599016).

All methods were carried out in accordance with relevant guidelines and regulations and are reported in accordance with ARRIVE guidelines for the reporting of animal experiments.

## Supplementary Information

Supplementary Information 1.

Supplementary Figures.

## Data Availability

The datasets used and/or analyzed during the current study are available from the corresponding author on reasonable request.
