# Supplementary material for: Anti-inflammatory effect of gold nanoparticles supported on metal oxides
Source: Sci Rep. 2021 Nov 30;11:23129. doi: 10.1038/s41598-021-02419-4 (PMC8632916; doi:10.1038/s41598-021-02419-4)
Supplement: Supplementary file 1 — Supplementary Information 1. [file 41598_2021_2419_MOESM1_ESM.docx]

**Supplementary information (SI)**

**Anti-inflammatory effect of gold nanoparticlessupported on metal oxides**

TakashiFujita^1,2*^, Maeva Zysman^3,4,5^, Dan Elgrabli^3,6^, Toru Murayama^1^,

Masatake Haruta^1^, Sophie Lanone^3^, Tamao Ishida^1^, Jorge Boczkowski^3,7*^

*(1) Research Center for Gold Chemistry, Department of Applied Chemistry for Environment, Graduate School of Urban Environmental Sciences, Tokyo Metropolitan University, 1-1Minami-osawa, Hachioji, Tokyo 192-0397, Japan*

*(2) DepartmentofAppliedChemistry, School of Engineering, Tokyo University of Technology,1401-1Katakura,Hachioji,Tokyo192-0982, Japan*

*(3) Univ Paris Est Creteil, INSERM, IMRB, F-94010 Creteil, France*

*(4) UnivBordeaux, Centre de Recherche cardio-thoracique de Bordeaux, U1045, CIC 1401, Bordeaux, France.*

*(5) Service des Maladies Respiratoires, CHU Bordeaux, Bordeaux, France*

*(6) SAS NaorInnov, Courbevoie, France*

*(7) AP-HP, Hopital Henri Mondor, Antenne de Pneumologie, F-94010 Creteil, France*

** corresponding authors:*[*fujitatks@stf.teu.ac.jp*](mailto:fujitatks@stf.teu.ac.jp)*,* [*jorge.boczkowski@inserm.fr*](mailto:jorge.boczkowski@inserm.fr)

**Methods**

**Phagocytosis assay**

RAW 264.7 cells (ATCC) were exposed to 50 μg·mL^-1^ofAu3/TiO_2_, Au8/TiO_2_or TiO_2_NPs for 6 h, together with Latex beads-rabbit IgG-Fluorescein 5-isothiocyanate(FITC) complex, as per the manufacturer’s instructions (Phagocytosis assay Kit; Cayman Chemical, 500,290). Fluorescence was measured at λexc = 485 nm, and λemi = 535 nm(1).

**Transmission electron microscopy (TEM)**

Macrophages submitted to different exposure were fixed overnight at 4 °C using TEM-grade fixative solution of 2% formaldehyde and 2.5% glutaraldehyde in 0.1 M sodium cacodylate buffer, pH 7.4. The samples were washed and stored in 0.1 M sodium cacodylate buffer and kept at 4 °C until processing. Sample embedding was performed in epoxy resin according a standard protocol.

1. Cohignac V, Landry MJ, Ridoux A, Pinault M, Annangi B, Gerdil A, et al. Carbon nanotubes, but not spherical nanoparticles, block autophagy by a shape-related targeting of lysosomes in murine macrophages. Autophagy. 2018;14(8):1323–34.
