# Supplementary material for: Anti-inflammatory effect of gold nanoparticles supported on metal oxides
Source: Sci Rep. 2021 Nov 30;11:23129. doi: 10.1038/s41598-021-02419-4 (PMC8632916; doi:10.1038/s41598-021-02419-4)
Supplement: Supplementary file 2 — Supplementary Figures. [file 41598_2021_2419_MOESM2_ESM.pptx]

## Slide 1
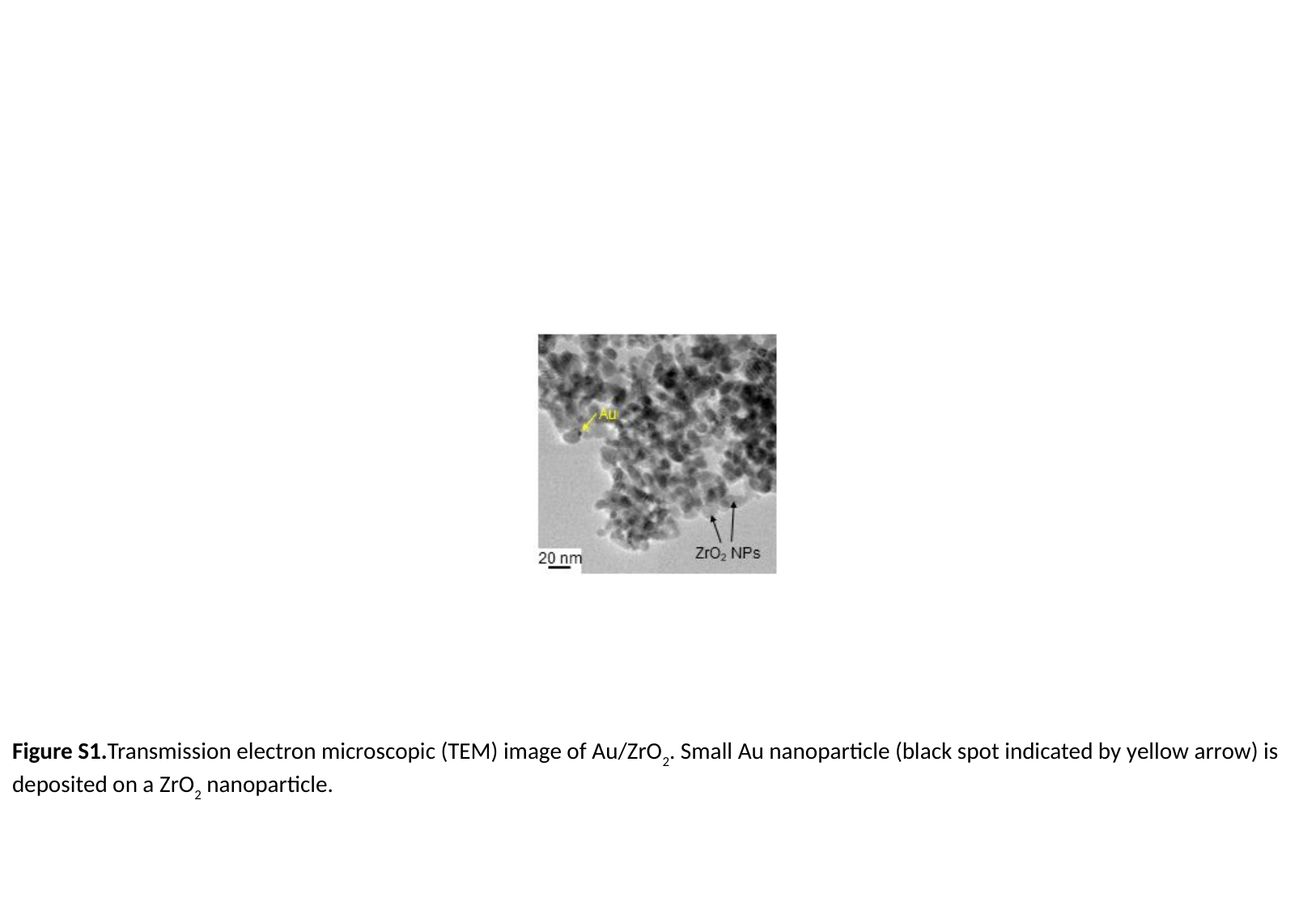

Figure S1.Transmission electron microscopic (TEM) image of Au/ZrO2. Small Au nanoparticle (black spot indicated by yellow arrow) is deposited on a ZrO2 nanoparticle.

## Slide 2
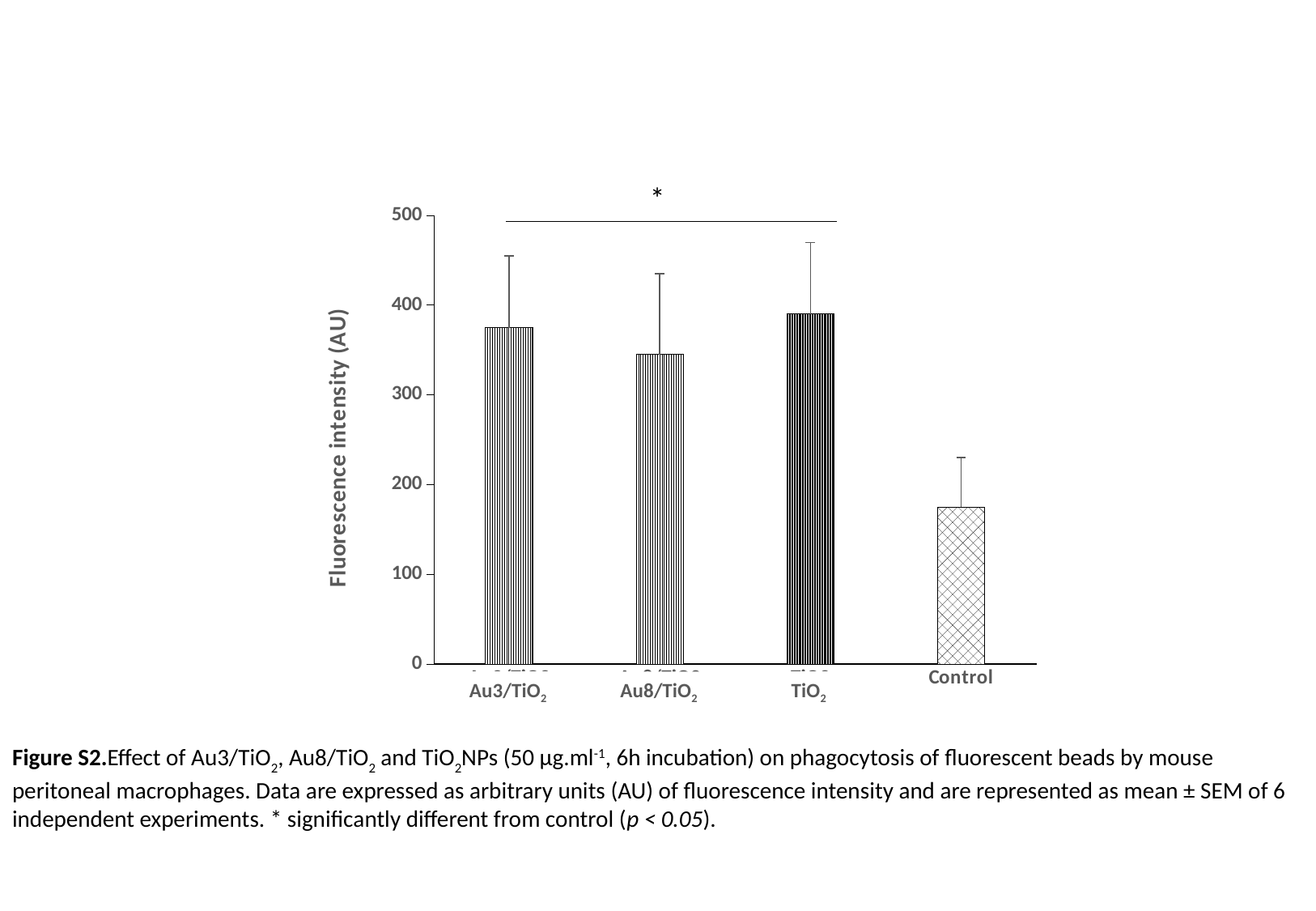

*
### Chart
| Category | |
|---|---|
| Au3/TiO2 | 375.0 |
| Au8/TiO2 | 345.0 |
| TiO2 | 390.0 |
| Control | 175.0 |Au3/TiO2
TiO2
Au8/TiO2
Figure S2.Effect of Au3/TiO2, Au8/TiO2 and TiO2NPs (50 µg.ml-1, 6h incubation) on phagocytosis of fluorescent beads by mouse peritoneal macrophages. Data are expressed as arbitrary units (AU) of fluorescence intensity and are represented as mean ± SEM of 6 independent experiments. * significantly different from control (p < 0.05).

## Slide 3
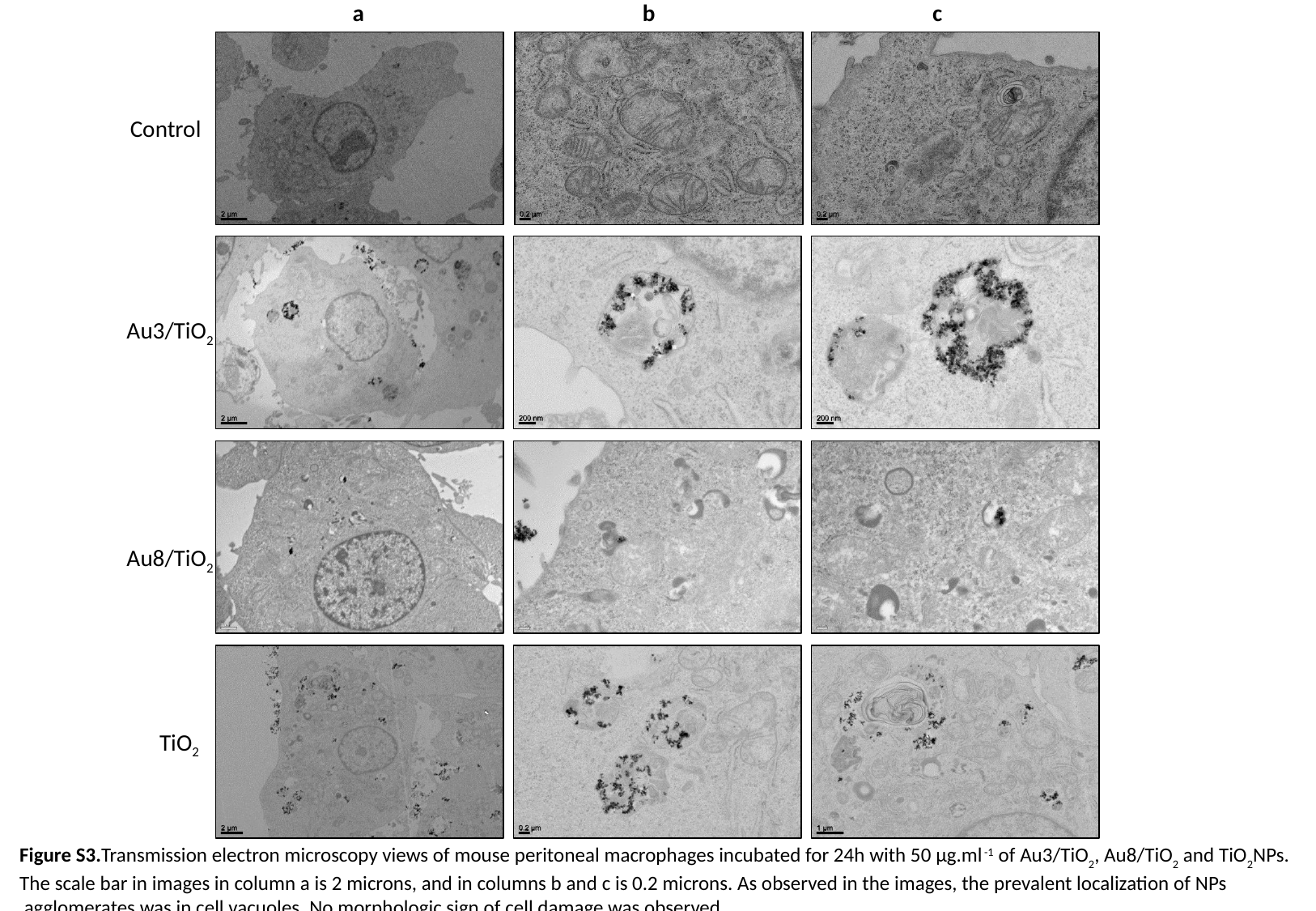

a
b
c
Control
Au3/TiO2
Au8/TiO2
TiO2
Figure S3.Transmission electron microscopy views of mouse peritoneal macrophages incubated for 24h with 50 µg.ml-1 of Au3/TiO2, Au8/TiO2 and TiO2NPs.
The scale bar in images in column a is 2 microns, and in columns b and c is 0.2 microns. As observed in the images, the prevalent localization of NPs
 agglomerates was in cell vacuoles. No morphologic sign of cell damage was observed.
